# Cholera Management and Prevention at Hôpital Albert Schweitzer, Haiti

**DOI:** 10.3201/eid1711.110815

**Published:** 2011-11

**Authors:** Silvia Ernst, Carolyn Weinrobe, Charbel Bien-Aime, Ian Rawson

**Affiliations:** Hôpital Albert Schweitzer, Deschapelles, Haiti

**Keywords:** bacteria, waterborne infections, cholera, water purification, sanitation, epidemic, Haiti, dispatch

## Abstract

In October 2010, Hôpital Albert Schweitzer Haiti treated some of the first patients with cholera in Haiti. Over the following 10 months, a strategic plan was developed and implemented to improve the management of cases at the hospital level and to address the underlying risk factors at the community level.

Hôpital Albert Schweitzer (HAS), Haiti, built in 1956, is a 130-bed, private, nonprofit facility that serves >340,000 persons in the Artibonite region of central Haiti. The mission of HAS is to reduce illness and death by providing primary and secondary health care, building wells and latrines, and supporting community development activities.

On October 19, 2010, HAS began to receive male patients without underlying medical conditions who had acute watery diarrhea and vomiting. This clinical presentation was unusual; the rapid onset and severity of dehydration was atypical of diarrheal diseases seen at the hospital. Three days later, on October 20, 2010, an additional 24 persons arrived with the same signs, causing alarm among medical personnel, who then contacted the Ministry of Public Health and Population (MSPP) and the Centers for Disease Control and Prevention (CDC, Atlanta, GA, USA).

Concomitantly, HAS received news that the densely populated city of St. Marc, ≈20 km to the west along the Artibonite River, had a large number of persons with similar signs. Although cases of cholera had not been documented in Haiti for over a century (*1*), health care providers began to suspect cholera because of the patients’ clinical signs. On October 21, 2010, the National Laboratory of Public Health officially announced a cholera outbreak in the Artibonite region. In the days after seeing the initial cases, HAS developed a strategic plan to 1) create an isolated patient area, 2) train medical personnel in cholera treatment, 3) educate the population, and 4) improve poor sanitary conditions through latrine building in the community.

## Management Strategies

A temporary isolated cholera treatment area was set up in the hospital’s courtyard, and routine hospital services were relocated to prevent nosocomial spread. MSPP, with the help of international organizations, established cholera treatment centers (CTCs) at 2 nearby public hospitals, while HAS acted as a referral hospital to treat immunosuppressed patients and patients with complicated cases. However, patients with cholera continued to seek care at HAS, which required the hospital to develop a longer term plan to manage cholera patients.

The medical director implemented the World Health Organization cholera treatment protocols, which included oral rehydration solution for patients with moderate dehydration and intravenous Ringer’s lactate solution and antimicrobial drugs for the patients with severe dehydration (*2*). All medical personnel received training in implementation of these protocols. Cases were defined as persons >5 years of age who were admitted to HAS with acute watery diarrhea, with or without vomiting.

Infection control was a top priority for HAS. Beds were removed and disinfected after each patient was discharged. With a limited number of nurses, family members were trained to be the primary caregivers for patients in the CTC; training included proper hygiene. Sanitation staff supervised handwashing stations, disinfected all buckets used for waste collection, disinfected the floors and walls, and sprayed the shoes of everyone exiting the facility with chlorinated water.

Although the initial cases were among men working in the rice fields, cholera quickly spread among persons of all age groups, both sexes, and residing in valley and mountain regions. A complicated disease course often developed in the elderly and those patients with underlying medical conditions. Older patients experienced renal failure, pulmonary edema, and heart failure; hypoglycemia developed in young children who were malnourished. Severe dehydration among pregnant women caused many to have miscarriages, a known side effect of cholera (*3*). These complications were challenging because patients could not be transferred from the CTC to the hospital for specialized care because of cholera’s contagiousness. Because medical records were incomplete for almost half of the patients with cholera, an accurate overall case-fatality rate could not be calculated. Among the 2,359 patients for whom outcome data were available, the case-fatality rate at the hospital was 0.8%.

**Figure 1:**
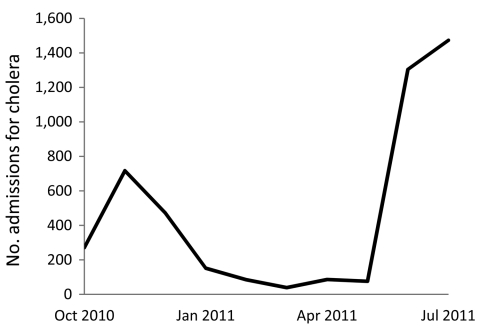
Cholera treatment center admissions, patients >5 years of age, Hôpital Albert Schweitzer, Artibonite Department, Haiti, October 17, 2010–July 31, 2011. N = 4,606. Data from cholera treatment center admissions records.

As the number of cases continued to rise in November 2010, HAS opened a permanent CTC adjacent to the hospital. By January 2011, there were only on average 6 new admissions per day to the CTC, so staffing was considerably reduced. At the same time, CTCs run by international organizations in neighboring towns were reducing operations and shifting management responsibilities to MSPP. In late May 2011, the number of CTC admissions increased rapidly with the beginning of the rainy season; by June 29, 2011, the number of CTC admissions peaked at 132 new cases per day, a rate even greater than that during the initial outbreak (Figure 1). With no other CTCs nearby operating 24 hours a day and able to treat persons with severe dehydration, HAS served a large geographic area.

**Figure 2:**
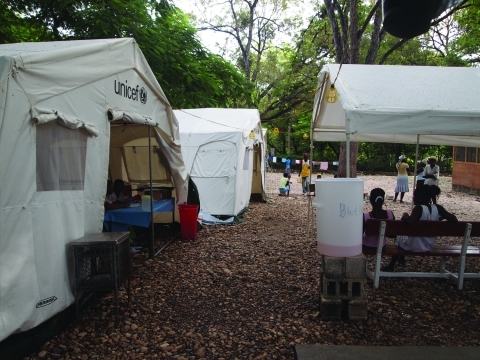
Tents where patients with cholera were treated at Hôpital Albert Schweitzer, Artibonite Department, Haiti, October 17, 2010–July 31, 2011.

During this outbreak, patients were treated in 6 tents organized by severity of dehydration (Figure 2). As the outside temperature was >90°F, the temperature inside the tents was much higher, posing a threat to already severely dehydrated patients. Patients were closely monitored by nurses and family members to ensure adequate hydration. The CTC’s waste management system was overloaded, so more latrines were built. Visiting teams of doctors and nurses supported the permanent staff in dealing with the patient surge.

Community mobilization was essential for slowing the spread of the outbreak. After initial reports that severe diarrhea was being treated with traditional remedies of guava leaves and rum and that persons were dying of “supernatural causes,” HAS staff began meeting with influential community members such as traditional healers, teachers, and religious and local leaders. Community health workers, trained by HAS, educated the community on cholera signs and symptoms and disseminated prevention messages on proper hygiene while also distributing oral rehydration solution and water treatment products.

## Conclusions

Poor sanitary conditions were present in the community long before the cholera outbreak (*4*). Although an informal survey of CTC patients from January 2011–May 2011 showed that most knew to drink treated water and wash their hands, only slightly more than half had access to a latrine. Soap and water-treatment products distribution addressed the acute but not chronic need for latrines. After site visits that included hygiene education and latrine assessments, HAS constructed 266 latrines. The hospital continues to work with regional partners to expand these activities.

Point-of-use water treatment has proven to be a more complicated intervention; powdered chlorine to add to water and charcoal for boiling water are expensive, and water purification tablets are donor driven and lack standardization across organizations. Different types of tablets treat different amounts of water, and low literacy rates and language comprehension make ensuring proper use of the tablets challenging.

After the surge in cholera cases in June 2011 with the start of the rainy season, it is likely that cholera has become endemic to rural Haiti. HAS is prepared to address the clinical consequences of this disease, but more needs to be done to reduce illness and death. The rise in cases signaled that the MSPP hospital infrastructure was not capable of addressing the disease independently. Without a regional plan that incorporated private and public institutions, cholera treatment was conducted primarily by HAS, a private institution. The lack of preparedness across the Artibonite region for the resurgence of cholera during the rainy season proved that a unified system must be fully equipped to manage future outbreaks. Such a system should be developed by MSPP in collaboration with private institutions and international organizations.

To reduce the effects of cholera in rural Haiti, all households should have access to latrines and clean water (*5*). To reduce cholera illness and death, private and public hospitals must work closely with the government and international organizations to standardize a nationwide plan of action.
